# Analysis of long branch extraction and long branch shortening

**DOI:** 10.1186/1471-2164-11-S2-S14

**Published:** 2010-11-02

**Authors:** Timothy O’Connor, Kenneth Sundberg, Hyrum Carroll, Mark Clement, Quinn Snell

**Affiliations:** 1 Department of Zoology, University of Cambridge, Cambridge CB2 3EJ, UK; 2 Department of Computer Science, Brigham Young University, Provo, Utah 84602, USA

## Abstract

**Background:**

Long branch attraction (LBA) is a problem that afflicts both the parsimony and maximum likelihood phylogenetic analysis techniques. Research has shown that parsimony is particularly vulnerable to inferring the wrong tree in Felsenstein topologies. The long branch extraction method is a procedure to detect a data set suffering from this problem so that Maximum Likelihood could be used instead of Maximum Parsimony.

**Results:**

The long branch extraction method has been well cited and used by many authors in their analysis but no strong validation has been performed as to its accuracy. We performed such an analysis by an extensive search of the branch length search space under two topologies of six taxa, a Felsenstein-like topology and Farris-like topology. We also examine a long branch shortening method.

**Conclusions:**

The long branch extraction method seems to mask the majority of the search space rendering it ineffective as a detection method of LBA. A proposed alternative, the long branch shortening method, is also ineffective in predicting long branch attraction for all tree topologies.

## Background

Due to its speed and simplicity, one of the most common methods used in phylogenetics is Maximum Parsimony [2] (MP). MP is based on the principle of the Occam’s razor, which means the simplest explanation for any phenomenon is the most probable. Under this principle parsimony makes the claim of using few if any assumptions, and while this has been disputed, MP’s model is much more simple with far fewer parameters than many other phylogenetic methods. Three major problems have been cited with MP, stemming from this assumption of simplicity. Many authors have argued that parsimony has under parameterized the problem, then the claim was made that it over parameterizes the problem [3]. The third problem is that of Long-Branch Attraction (LBA).

LBA is the foundation for many of the arguments against the use of MP in phylogenetics. One foundational study showed that MP can be positively misleading when two non-sister taxa have long branches compared to the rest of the tree [4]. This bias has then been reiterated in a number of other simulated and empirical studies (see Bergsten [5] for an in-depth review of the current debate on LBA). The crux of the problem is that long branches, whether sister taxa or not, are claded or grouped together, creating scenarios where the MP method will consistently be incorrect. This has the potential to occur often, when given enough evolutionary time because multiple sites will differentiate from each other. Since there is a finite set of characters, (i.e. A,C,G,T for DNA) the two sequences will have many sites with matching characters. As more evolutionary time passes, fewer of these sites will be due to a common ancestor or homology, and more of them will be due to the random use of the same nucleotide. This non-homologous yet similar sequence of characters adds noise to the phylogenetic signal. This problem is not unique to parsimony but parsimony suffers from it more extensively than Maximum Likelihood (ML) [6-10].

LBA has been found in many real world examples, one review found 112 examples in a search on the Web of Science [5]. This illustrates the need for a method that can accurately evaluate if a phylogenetic analysis is suffering from LBA. Some methods have been designed in an attempt to fill this gap including: “methodological disconcordance, RASA, separate partition analysis, parametric simulation, random outgroup sequences, long-branch extraction, split decomposition and spectral analysis.” [5] Many of these current methods have been shown to be ineffective or reliant on morphological data. Reliance on morphological data is effective but creates problems due to the difficulty in gathering and sampling this kind of data, so many researchers rely exclusively on molecular data. Long Branch Extraction (LBE), also referred to as Long Branch Abstraction, was developed by Siddall, Whiting, and Pol as a method to detect LBA. LBE relies on the assumption that if there are two long sequences, removing one of them should move the other to its correct place in the tree, and if this location is different than its original location, the dataset suffers from from LBA [1,9]. In [5] Bergsten proposes a six step method to detect LBA based on the LBE method. These steps are:

1. If, after completing a full parsimony search you obtain a tree with a questionable grouping of a certain taxa that appears basal and makes the formal classification polyphyletic, suspect LBA.

2. Exclude the outgroup and re-run the analysis: does the questionable taxa form a monophyletic clade of the formal classification?

3. Return the outgroup and remove the questionable taxa and re-run the analysis: does this root the tree differently then in step 1 (later compare to step 4 and 5 as well)?

4. Return the questionable taxa and reanalyze the data set by separating the gene information from the morphological data: does the morphological data form a monophyletic group of the formal classification while the gene data place the questionable taxa basal in the tree?

5. Analyze the gene data using a method that takes into account branch lengths, (i.e. Bayes or Likelihood): does this method form a monophyletic group of the formal classification?

6. Using the same analysis of step 5: are the branch lengths of the questionable taxa and the outgroup some of the longest in the tree?

If you can answer yes to all the previous questions, LBA is the least refuted hypothesis. We have chosen to automate this technique with a few modifications and evaluate it on a series of synthetic data with six taxa under a variety of branch lengths with verified LBA.

The six taxa synthetic data sets were used for two main reasons. Six taxa data sets are small enough to be calculated in reasonable time but large enough for the LBE method to work. This gave us an *a priori* knowledge as to which trees were suffering from long branch attraction.

## Methods

### Synthetic data sets

**Figure 1:**
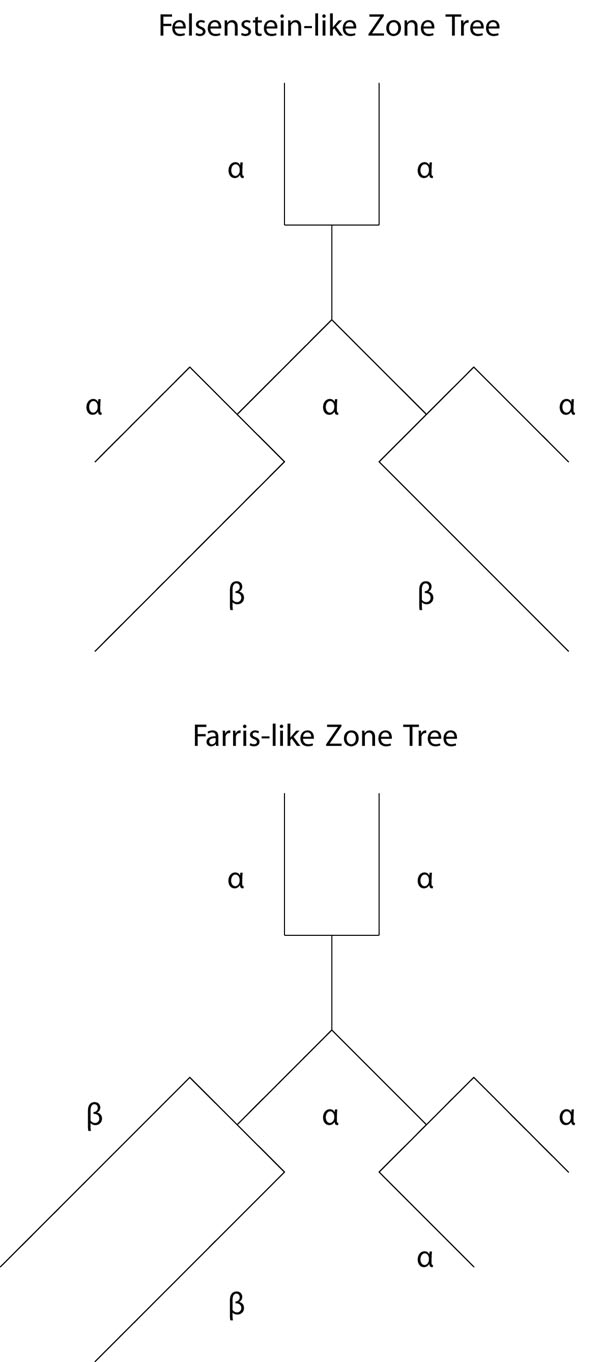
**The Felsenstein-like and Farris-like topologies used to simulate the data.** The six taxa star shape Felsenstein-like and Farris-like topologies.

To evaluate long branch extraction, datasets that allow for extraction of long branches and differentiation of topological location are necessary. We chose to perform the analysis using six taxa in a star shape for consistency and comparability to the more prevalent studies using four taxa cases of the Felsenstein and Farris (or reverse-Felsenstein) zone topologies [6,11] (see Figure 1). These scenarios present problems to phylogenetic methods because of the challenge to some assumptions they make. For example, parsimony assumes similar characters to be derived from a common ancestor, but with long non-sister branches there is a great probability that the two sequences are really analogous, meaning they have converged to the same character independently. Parsimony generally puts longer branches together in a four taxa case and here the same or similar problems have been preserved.

To produce these data sets we used the program Dawg [12] under a General Time Reversible (GTR) [13-15] model of evolution. We used similar parameters as those found in the examples included with the program and explored a range of branch lengths. The lambda value of 0.1 was used for the indel evolution rate and can be interpreted as one indel for every ten substitutions. The sequence length was set to 2000 as this gives a reasonably sized sequence to allow for the expected value of any simulation to be seen. The nucleotide frequencies for the simulation were set to 0.2, 0.3, 0.3, and 0.2 for A, C, T, and G respectively with substitution parameters set to 1.5, 3.0, 0.9, 1.2, 2.5, and 1.0 for AC, AG, AT, CG, CT, GT respectively. These settings were chosen based on examples given with the Dawg program.

Dawg generated data sets for trees under both topologies where the *α* and *β* branch lengths were varied from a branch length of 0.1 to 2.0, incremented by 0.1. A branch length of one is interpreted to mean that each site is expected to have one substitution from the internal node under the GTR definition of branch length. For each permutation of *α* and *β* branch lengths we ran 100 replicates to get a percentage of matches between the two methods. This created a total of 40,000 data sets for each topology.

### Evaluation of LBA area

Each data set was then analyzed by comparing the best parsimony tree from an exhaustive search. With six taxa this means scoring all 105 possible trees to find the best one. This best tree was then compared to the parsimony tree and the percentage of the trials out of 100 that the two matched was recorded. Then for each of the permutations of *α* and *β* we generated a new set of 100 data sets and performed a heuristic TBR parsimony search. All scoring and searching was done with PAUP* [16]. The tree that MP returned from the heuristic search was then analyzed using LBE.

### Steps of LBE

To perform LBE, the target tree, in our case the resultant heuristically derived parsimony tree, and data set are given as parameters along with a list of outgroup taxa and questionable taxa to our Java version of LBE. Of the two *β* branches, one was selected as the questionable taxa while the other was selected as the outgroup.

The first step of LBE is to remove the outgroup from the tree and the data set and rerun a parsimony search. The second step is to add the outgroup back and remove the questionable taxa. To increase the sensitivity, according to the recommendations of Bergsten (see “Concluding discussion: suggestions” from [5]) we included a third step where the original data set was evaluated under a branch length estimator method. We used Maximum Likelihood, and the resultant tree was compared to the original parsimony tree. If at any step the tree found by the re-ran search is the same as the original tree, minus the removed taxa in the first two steps, then LBA is no longer suspected and the search is terminated. If instead it passed through all of the steps, the branch lengths of the outgroup and the questionable taxa were compared to the rest of the branch lengths. If they were in the top quartile they were considered long branches. Having passed through each step or test, the least disputed hypotheses based on molecular data would be LBA.

## Results and discussion

### Areas under LBA

**Figure 2:**
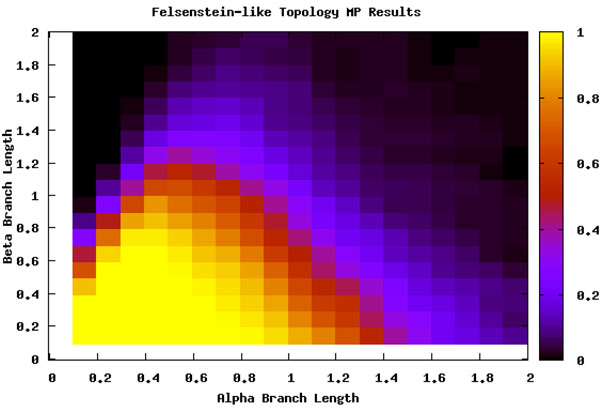
**Maximum Parsimony and the Felsenstein topology** Percentage of runs that MP identifies the true tree under the Felsenstein-like topology. Trees found in the upper left corner (the dark black area) suffer from LBA. The dark outer edge is where the signal is lost from too long of branches.

**Figure 3:**
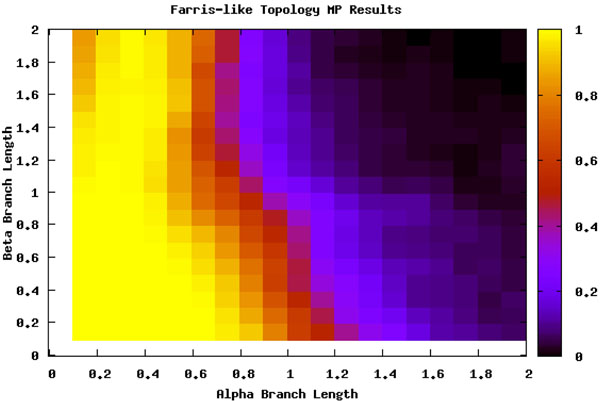
**Maximum Parsimony and the Farris topology** Percentage of runs that MP identifies the true tree under the Farris-like topology. Note the large area predicted correctly by MP; this is the area where the sister taxa have long branches and are correctly placed together based on MP’s bias.

To detect the areas most effected by LBA, we ran an analysis of the six taxa data sets (see section ) over a range of branch lengths and with two scenarios for the position of the long branches (see Figure 1). Figure 2 shows where the location of LBA, as the black region when the *β* branches are long and the *α* branches are shorter under the Felsenstein-like topology. As a control, the Farris-like topology shows how the parsimony bias can be perceived as increased accuracy under the same permutations of branch length. In these figures, the darker the color means the less amount of time the MP analysis and the correct topology were in accordance. In other words, the yellow areas are regions where MP always returned the correct topology (i.e. 100 out of 100 trials) and the black areas are where MP never returned the correct topology. The gradient obviously then covers the percentage of time at intermediate levels of accuracy. What is also interesting to note is the extreme cut off between the areas of correct prediction and those that are incorrect, especially when examining the Felsenstein-like topology of Figure 2. This very black region essentially shows the Felsenstein zone or the conditions under which parsimony suffers from LBA. Figure 3 shows the results for Parsimony under the Farris topology.

One problem with most phylogenetic algorithms is the loss of detectable signal with extremely long trees. The length of the tree is the sum of all the branch lengths it has and those with an extreme length or long trees are difficult to decipher. This problem is clearly visible when examining the upper right of the figures under both topologies. We hypothesis that as the branch lengths get longer the percentage correct will converge to 0.95% as this is a random guess out of the 105 possible topologies.

**Figure 4:**
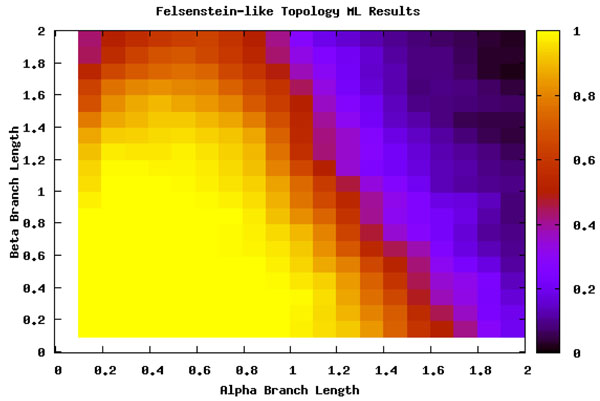
**Maximum Likelihood and the Felsenstein topology** Percentage of runs that ML identifies the true tree under the Felsenstein-like topology. Notice that the area of high accuracy is much larger and covers most of the LBA region. ML is not as susceptible to LBA.

This analysis served as a search space basis for where LBA should be detected. By comparing the differences between the Felsenstein-like and Farris-like topologies it is clearly visible which areas should be detected. When analyzed with ML these regions do not appear but the loss of signal is still present (see Figure 4). The comparison of both the topologies and the ML method adds further descriptive details and confidence to the search space we are examining.

### LBE is not functioning as theory predicts

For a method to accurately detect LBA, it needs to discern between these two types of topologies and find the area of LBA. The region found by searching the branch length space should be the same predicted by LBE. Surprisingly this was not the case.

**Figure 5:**
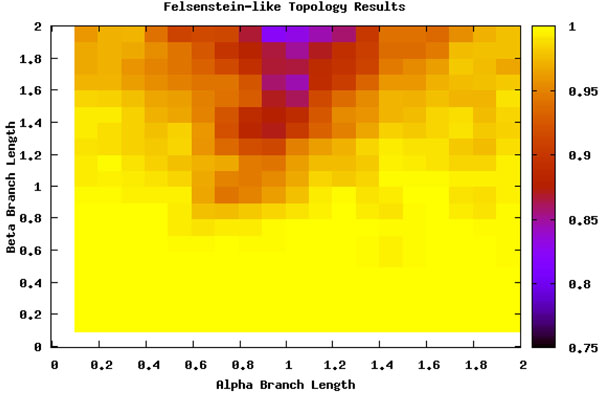
**Long Branch Attraction and the Felsenstein topology** Felsenstein-like topology. The color gradient represents the percentage of trees that were not predicted to have LBA.

**Figure 6:**
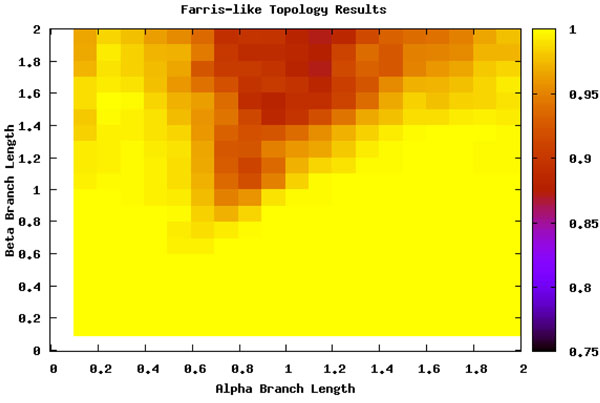
**Long Branch Attraction and the Farris topology** Farris-like topology. There should be no detection of LBA in this scenario because the long-branches are sister taxa.

As is seen in Figures 5 and 6, LBE seems to completely miss the area it is intended to detect (the upper left corner). For a more in depth investigation we analyzed the data by examining specific scenarios that should show extreme LBA. In the majority of cases examined, the parsimony trees outputted in the LBA zone really did suffer from LBA as predicted, but the method failed to recognize it and the short sister taxa of the removed taxa was incorrectly grouped with the other long branch.

Further, LBE predicted LBA under the Farris-like topology, where we know *a priori* that the data set does not suffer from LBA. A few inconsistent categorizations would be understandable because no method is perfect. But this situation, where similar branch lengths give similar conservative predictions under both topologies, calls into question what the method is actually predicting.

It is consistently classifying the wrong area of the Felsenstein-like topology as LBA and the same area of the Farris-like topology. In reality, this is an area suffering from loss of signal. But even in other areas of loss of signal, i.e. the lower right corner of Figure 2, it is classifying it as not having any LBA. Even though this is technically correct the loss of signal should produce a random-like result in the prediction of LBA, not an extremely confident vote that it is not suffering from LBA. Keeping in mind the method is detecting LBA as the least refuted hypothesis, it seems odd that the only area detected as having LBA is not actually suffering from it and those areas that are suffering from LBA have inconsistent results.

**Figure 7:**
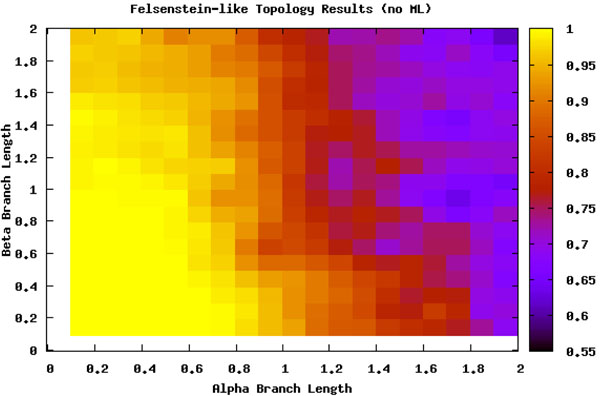
**Long Branch Extraction without branch length estimation** Felsenstein-like topology. This was a reanalysis without the third step, which looks at a branch length estimator ML. The detection is further biased and the area with less signal is confused with LBA.

What is more bothersome is that the LBE does not seem to consistently categorize based on specific examples of branch length. Under the full method of LBE with the branch length step included (see section ), the method only categorizes a maximum of 25% of any permutation of *α* and *β* as suffering from LBA. When the steps that use branch length estimation (i.e. ML) are removed, the LBE method categorizes more areas with a greater percentage of LBA, (45% in Figure 7) but looses its conservative nature with respect to areas that have lost signal. In this case, it inaccurately predicts a large area that had previously been defined as having lose of signal as having LBA be the least refuted hypothesis.

### Why it may not work

Siddall and Whiting make the claim that, “... if each of the two branches individually group in precisely the same place as the other when they are allowed to stand alone in an analysis, one can hardly argue that they are attracted to this placement by the absent branch. [1]” While this seems logical, one needs to remember that a common way to avoid LBA in the first place is to add additional taxa to break up long branches [17,18]. One possible reason that extracting taxa doesn’t work to detect LBA is that parsimony is sensitive to the removal of taxa, creating artificial long branches in the reran analysis. In the case of our analysis, removing a taxa would still be classified as not LBA because it created an artificially long branch consisting of a full *α* branch along with a half *α* branch. This then would attract either the original long branch taxa and it would look the same as the original LBA tree and then be rejected as LBA. In other words the extraction creates a problem with sampling, not splitting up longer branches by adding taxa, a typical pitfall when dealing with LBA. The long branch is not being attracted by the excluded long branch but it is being attracted to the extended branch caused by not breaking it up. This creates a double error and deceives the procedure into thinking it is not a case of LBA

**Figure 8:**
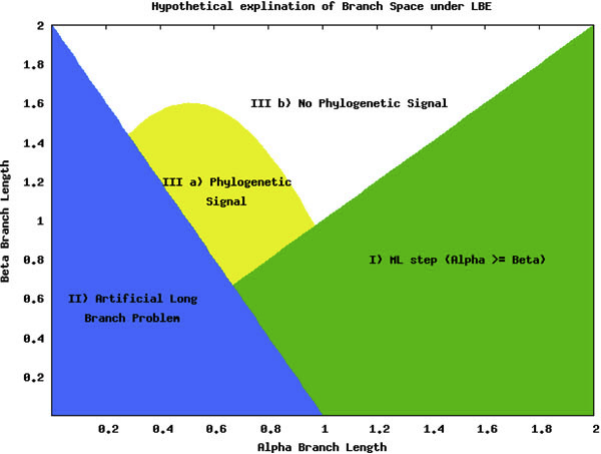
**Hypothetical explanation of branch length search space** Hypothetical explanation of branch length search space. I) This area is caused by the ML step predicting the *β* branches not being in the highest quartile, the top 25%. This is caused by *α ≥ β.* II) This area is created as a result of the artificial long branch created by extracting a taxa. This also masks the area important for LBA. IIIa) This area still has phylogenetic signal but is unambiguously not LBA. It is correctly identified by LBE but is not an area of interest. IIIb) The phylogenetic signal in this area has been lost to both MP and ML.

We can thus split the branch length search space into three major areas: the area masked by the ML step (I), the area misled by the artificially long branch (II), and the area that is correct until it reaches a point of loss of signal (III), as seen in Figure 8. Area I can be seen by comparing Figure 5 and Figure 7. The deciding factor when the branch lengths are *α ≥ β* is the final step that estimates the percentile of the outgroup and questionable taxa are among the top 25%. But we know, based on the design of our experiment, that this will not be the case in this area and so the detection or confusion that it is LBA is masked artificially. This mask is removed when we remove this final step from the analysis, as is seen in Figure 7 and the area looks like a continuation of a loss of signal area.

Area II is much more hypothetical but seems to fit the data reasonably well. When examining Figures 5 and 6 there is a noticeable but rough line at about *y = –*2 * *x* + 2. We hypothesis that the shape of this line is a function of the branch lengths. This area is obviously crucial as seen in Figure 5 because it is the area suffering from LBA. In other words, the predictive power of LBE is being masked by this artificial long branch in the exact area needed for accurate prediction of LBA. This triangle directly corresponds to the areas under LBA, thus making the technique inadvisable.

Finally, area III is where the LBE method is actually mostly correct or the area not suffering from some other artifact. Unfortunately, this area is not suffering from LBA but eventually it losses phylogenetic signal. It is the most clearly seen in Figure 4 where ML can determine to a greater extent the phylogenetic signal. At approximately the same point LBE makes incorrect predictions because of the loss of signal. This area is not under a LBA bias for MP and so is correctly labeled as not having LBA but this is not informative. This really does not add a lot of strength to the procedure because it is already unambiguous.

### Long branch shortening

Due to the problems associated with Long Branch Extraction, an alternate approach could be used. Rather than removing the suspected long branch that would cause changes in the overall phylogeny, a series of iterative steps are taken to shorten the branch to diminish the phylogenetic signal being sent from the questionable branch and then see if that changes the phylogeny. If the phylogeny changes, long branch attraction is suspected.

**Figure 9:**
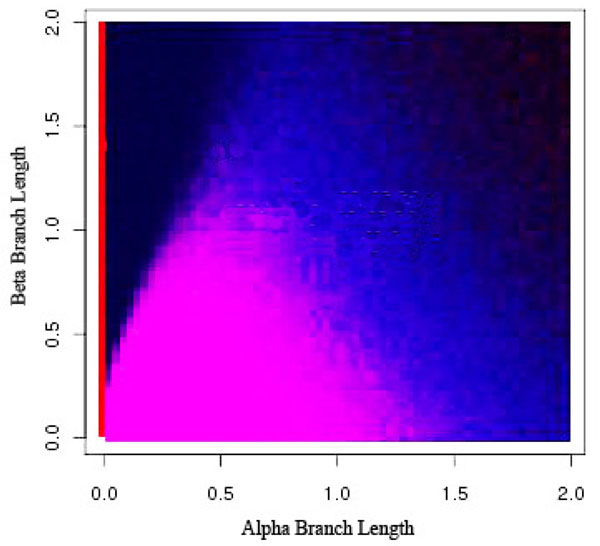
**Long Branch Shortening using 0% Sampling Frequency** Long Branch Shortening using different sampling frequencies. Black indicates an incorrect diagnosis from LBS while gray indicates LBS successfully determined whether or not long branch attraction was present in the resulting phylogeny.

**Figure 10:**
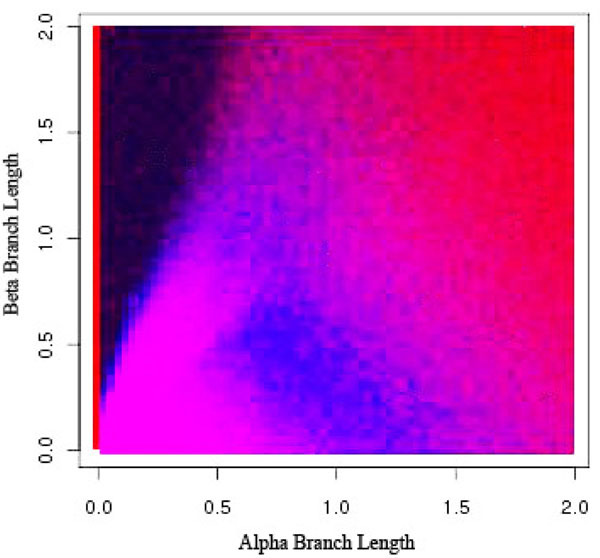
**Long Branch Shortening using 30% Sampling Frequency** Long Branch Shortening using different sampling frequencies. Black indicates an incorrect diagnosis from LBS while gray indicates LBS successfully determined whether or not long branch attraction was present in the resulting phylogeny.

**Figure 11:**
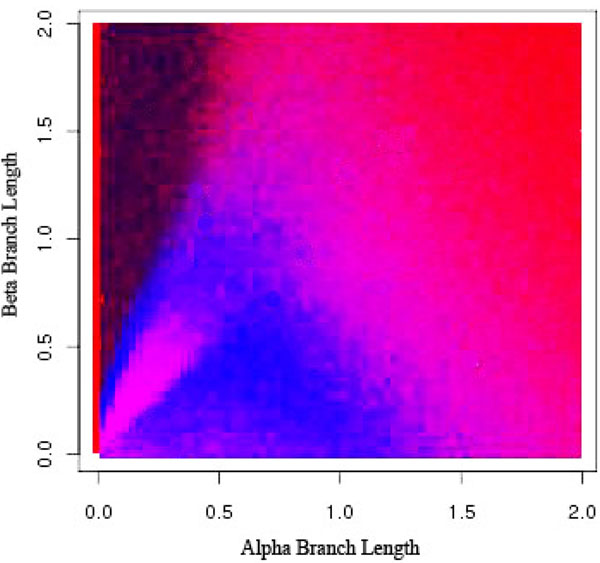
**Long Branch Shortening using 60% Sampling Frequency** Long Branch Shortening using different sampling frequencies. Black indicates an incorrect diagnosis from LBS while gray indicates LBS successfully determined whether or not long branch attraction was present in the resulting phylogeny.

**Figure 12:**
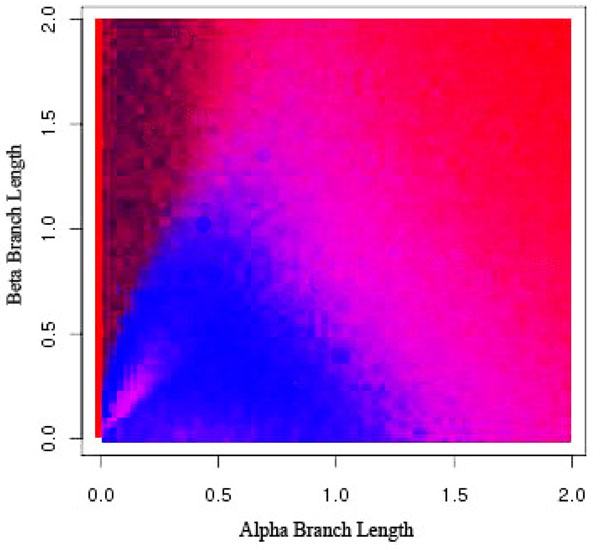
**Long Branch Shortening using 90% Sampling Frequency** Long Branch Shortening using different sampling frequencies. Black indicates an incorrect diagnosis from LBS while gray indicates LBS successfully determined whether or not long branch attraction was present in the resulting phylogeny.

Assuming the questionable taxon (qtaxa) falls basal in the MP analysis and is suspect (this is similar to step 1 of LBE), LBS performs the following three step test:

1. Rather than sampling from all the other taxa, construct the ancestral sequence to all taxa excluding the outgroup and qtaxa. With this sequence, you have the combined signal of all the other taxa, or a summary of that clade.

2. Using the constructed sequence and the questionable taxa, hybridize the two in a random fashion. We are not implying crossing over, albeit that should be tested as well, but using a binomial distribution, characters are exchanged between the sampled ancestor and the suspected long branch ataxa. This causes the branch to be shortened by reducing the differences between the taxon and its hypothetical ancestor. However, since this ancestor is unknown, the characters for the questionable taxon are modified by sampling from the hypothetical ancestor. In Figures 9, 10, 11, 12, different sampling frequencies were used. A sampling frequency of 30% means that a random 30% of the target taxon characters are modified.

3. Re-run the analysis with the hybridized sequence included in place of the qtaxa. If the taxa moves after reducing its own signal and adding some signal from the monophyletic clade you have some evidence of LBA The parameter or probability of switching in the binomial distribution is increased and steps 2 and 3 are repeated until either the probability reaches 1 or consistently (i.e. multiple runs) shows the hybridized qtaxa clading with the hypothetical clade.

One of the weaknesses of such an approach is the lack of an absolute answer. You don’t get a final answer of yes or no (as to whether LBA is occurring) but added evidence that there is a problem. This evidence comes in the form of a probability or percentage of the branch that needs to be shortened to form the monophyletic clade. If the probability comes out high, 0.9 to 1.0, you can be fairly sure that LBA is not occurring that that there is strong phylogenetic signal supporting the current position in the phylogeny. If it is very low, then long branch attraction has occurred and is causing an incorrect tree to be inferred. This evidence can help the researcher to understand if the questionable taxa (qtaxa) is sending a strong signal to be in the current location or a weak one. A weak signal implies that the location is inferred only because of analogous evolution and not homology. This implication can then be interpreted as the determination or detection of LBA.

In Figures 9, 10, 11, 12, dark black indicates regions where Long Branch Shortening(LBS) fails to predict whether long branch attraction is occurring. Gray areas indicate regions where LBS successfully determined whether or not long branch attraction was present in the resulting phylogeny. With a 0% sampling frequency (Figure 9), the target taxon is not modified at all and thus the phylogeny does not change. LBS then reports that no LBA exists anywhere. In this case, the Felsenstein Zone (the black region in the upper left portion of the graph) is clear and LBS is unable to detect long branch attraction. As the sampling frequency increases, the target taxon becomes more like the clade and LBS is more able to detect long branch attraction in the Felsenstein Zone. However, this comes at a price. The region where there is no long branch attraction is now reported incorrectly (the lower left portion of the graph). This is due to the fact that the target taxon has become so much more like the other taxa that at 90% sampling (Figure 12), long branch attraction is always reported because the target taxon always moves; resulting in a different phylogeny.

### Maximum parsimony and maximum likelihood

**Figure 13:**
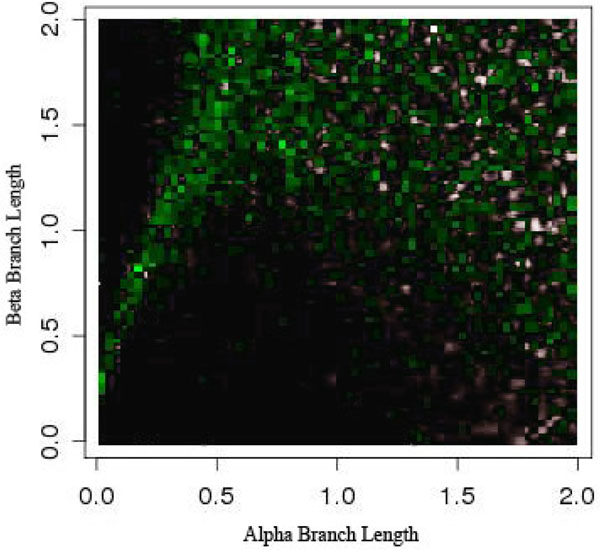
**Parsimony vs. Likelihood using 4 taxa** Comparison of Parsimony and Maximum Likelihood using a 4 taxa Felsenstein Zone tree.

**Figure 14:**
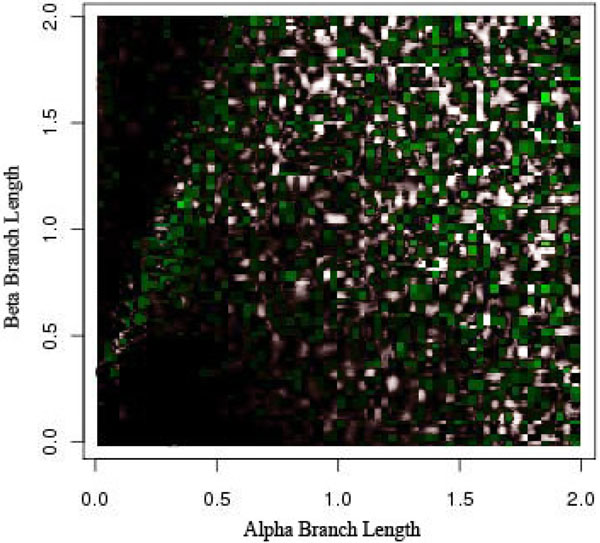
**Parsimony vs. Likelihood using 6 taxa** Comparison of Parsimony and Maximum Likelihood using a 6 taxa Felsenstein-like Zone tree.

Lastly, as this paper addresses algorithms to detect regions where Maximum Parsimony would report the incorrect tree, it is important to compare Maximum Parsimony and Maximum Likelihood in terms of the best scoring tree versus the true phylogeny. Figures 13 and 14 address this question. In the figures, dark areas indicate topologies where Maximum Likelihood and Maximum Parsimony generated the same phylogeny. In the gray regions, Maximum Likelihood generated the correct phylogeny while Maximum Parsimony failed and in the white regions, Maximum Parsimony generated the correct phylogeny and Maximum Likelihood failed. This shows that in the region where there is not much phylogenetic signal (the upper right region) both methods are equally likely to generate the correct tree. In the region near the Felzenstein Zone, Maximum Likelihood is able to generate the correct phylogeny but only for a small additional part of the region. Much of the Felsenstein Zone is still unable to be determined in both the 4 and 6 taxa cases by either Parsimony or Likelihood.

## Conclusions

Long Branch Extraction(LBE) and Long Branch Shortening(LBS) are not reliable methods for detecting Long Branch Attraction(LBA) and should not be used in phylogenetic inquiries about LBA. Under a variety of branch lengths for six taxa synthetic data sets LBE incorrectly and inconsistently predicts LBA because of its inability to distinguish between artificially created long branches and the correct tree topology. The artificial long branch is created by the removal of the outgroup or questionable taxa branch creating a sister taxa that is artificially long, having removed the taxa that would break up its long branch. An additional problem is that the ML step masks a large area of the branch length space not giving the method the specificity that is needed to be effective. This was shown by an in depth search over two topologies, the Felsenstein-like topology that is easily susceptible to LBA and the Farris-like topology in which the long branches are correctly grouped together. The results support our conclusion that LBE is ineffective in detecting LBA.

LBS is not effective because it incorrectly estimates the sequence present at the ancestral node. Statistical sampling of the other sequences artificially causes the target taxon to appear like all the taxa rather than shortening its branch. This results in a loss of accuracy in the detection of LBA.

Both LBE and LBS suffer from a secondary effect. When a branch is extracted from the phylogeny or shortened, other branches are free to become the longest branch and will potentially draw other similarly long branches away from their correct locations. Both Maximum Likelihood and Maximum Parsimony are subject to LBA in Felsenstein topologies and Likelihood provides superior results in only a small part of the Felsenstein Zone.

## Competing interests

The authors declare that they have no competing interests.

## Authors’ contributions

All authors designed, analyzed, implemented and tested the proposed algorithm. Each author contributed equally in writing the paper. All authors read and approved the final manuscript.
